# Surgical Management of an Anterior Mandibular Botryoid Odontogenic Cyst With GBR: A Case Report

**DOI:** 10.1155/crid/5297729

**Published:** 2026-07-14

**Authors:** Priscilla Sergi, Subhi Tayeb, Carlo Barausse, Martina Sansavini, Gerardo Pellegrino, Pietro Felice

**Affiliations:** ^1^ Unit of Oral Surgery, Department of Biomedical and Neuromotor Sciences, University of Bologna, Bologna, Italy, unibo.it

**Keywords:** case reports, cone beam computed tomography, differential diagnosis, enucleation, GBR, mandible, odontogenic cyst

## Abstract

**Background:**

The botryoid odontogenic cyst (BOC) is a rare multilocular variant of the lateral periodontal cyst (LPC), characterized by a more expansile growth pattern despite its benign nature. Whereas the LPC exhibits a very low recurrence rate, the botryoid variant is associated with a higher risk of recurrence, mainly due to its multilocular architecture. Since its first description in 1973, fewer than 100 cases have been reported in the literature.

**Case Presentation:**

A 40‐year‐old male patient, a smoker, with no significant systemic medical history, was referred for evaluation of a radiolucent lesion located between Teeth 43 and 44, incidentally detected on panoramic radiography. Complete surgical enucleation of the lesion was performed with preservation of the involved teeth, which underwent endodontic treatment and apicectomy. Given the CBCT and intraoperative evidence of buccal cortical plate loss and reduced defect containment, a selective guided bone regeneration (GBR) approach was used as an adjunct to support defect healing and soft tissue stability. Clinical and radiographic follow‐up at 6, 12, and 24 months reported complete healing and no evidence of recurrence.

**Conclusions:**

Complete surgical enucleation represents the treatment of choice for BOCs. Early diagnosis and long‐term radiographic follow‐up are essential to minimize the risk of recurrence and to ensure a favorable clinical outcome. The adjunctive GBR should not be considered routine but may be considered on an individual basis in selected defects with cortical plate loss or reduced bony containment.

## 1. Introduction

The botryoid odontogenic cyst (BOC) is a rare multilocular variant of the lateral periodontal cyst (LPC), recognized by the World Health Organization as a nonkeratinized epithelial odontogenic lesion. First described by Weathers and Waldron in 1973 [1], it is currently included in the *World Health Organization Classification of Head and Neck Tumours* among the cysts of the jaws [2]. The term “botryoid” derives from the Greek *botrys*, meaning “a cluster of grapes,” referring to the characteristic multilocular architecture of this entity [3, 4]. Whereas the LPC accounts for approximately 0.4%–1% of all odontogenic cysts, the botryoid variant represents an even smaller proportion [5, 6]. Since its initial description in 1973, fewer than 100 cases have been reported in the literature, underscoring the rarity of this lesion. Although its biological behavior is generally benign, the multilocular configuration and presence of internal fibrous septa are thought to confer a greater growth potential compared with the classic LPC. BOC occurs more frequently in the mandible rather than in the maxilla, with a predilection for the canine–premolar region [7]. It is rarely observed in pediatric or young patients and is typically diagnosed between the IV and VI decades of life. The etiology and histogenesis of BOC remain uncertain. Early hypotheses suggesting an origin from the epithelial rests of Malassez or the reduced enamel epithelium have largely been dismissed. Current evidence supports dental lamina remnants as a more plausible source, based on the histologic similarities between these structures and the cyst lining, both characterized by glycogen‐rich clear cells [8]. Histologically, BOC exhibits a multilocular cystic cavity lined by nonkeratinized squamous epithelium, frequently showing focal epithelial thickenings, plaque‐like proliferations, and multiple fibrous septa [9]. Clinically, the lesion is mostly asymptomatic and may be detected incidentally during routine radiographic examinations. In more advanced cases, it may cause displacement of adjacent teeth, vestibular swelling, or, less commonly, tooth mobility [3]. Panoramic radiography is typically the initial diagnostic modality; however, cone beam computed tomography (CBCT) is essential for evaluating the three‐dimensional extent of the lesion and its relationship with adjacent anatomical structures. Radiographically, BOC presents as a well‐defined radiolucency, occasionally containing internal septa responsible for the characteristic “grape‐like” appearance [10]. Although BOC is classically regarded as a multicystic variant of LPC, its radiographic presentation is variable and may be either unilocular or multilocular; therefore, radiographic appearance does not always mirror the underlying microscopic architecture. The radiographic presentation may overlap with those of several odontogenic and nonodontogenic lesions, including odontogenic keratocyst, unicystic ameloblastoma, central giant cell granuloma, ameloblastic fibroma, odontogenic myxoma, intraosseous mucoepidermoid carcinoma, and central odontogenic fibroma [3]. Consequently, histopathological examination is mandatory to establish a definitive diagnosis and to exclude more aggressive or recurrent entities. According to the WHO Essential Diagnostic Criteria, BOC is defined by (1) a location on the lateral aspect of, or between, the roots of vital erupted teeth, typically in the mandibular cuspid–premolar region, (2) the presence of distinctive whorled epithelial plaques, and (3) a multilocular architectural pattern [2]. Complete surgical enucleation is considered the standard treatment [1, 11]. Nevertheless, due to its multilocular nature and reported recurrence in selected cases, long‐term radiographic follow‐up is recommended [1, 12]. This case report is aimed at contributing to the limited body of literature on BOC by describing the clinical, radiographic, surgical, and histopathological features of an anterior mandibular presentation.

## 2. Case Presentation

The patient was treated at the Unit of Oral Surgery, Department of Biomedical and Neuromotor Sciences (DIBINEM), University of Bologna, Italy. The objectives and procedures related to this case report were thoroughly explained to the patient, and written informed consent was obtained. The manuscript was prepared in accordance with the CARE (CAse REports) [13] guidelines to ensure completeness and transparency in clinical reporting. All procedures were conducted in accordance with the Declaration of Helsinki, and ethical approval was obtained from the Ethics Committee of the Azienda Unità Sanitaria Locale (AUSL) Città di Bologna, Italy. A 40‐year‐old male patient, smoker (approximately 20 cigarettes per day), in good general health but with a remote history of pulmonary tuberculosis, presented to his general dentist for a routine dental checkup. A panoramic radiograph obtained during the routine examination revealed a well‐defined unilocular radiolucent lesion located in the interradicular area of Teeth 43 and 44 (mandibular right canine and first premolar). The patient was subsequently referred to the Oral Surgery Unit for further evaluation. The patient reported a mild subjective sensation of progressive displacement of the mandibular anterior teeth but denied pain, swelling, or other associated symptoms. Intraoral examination revealed a vestibular swelling in the region of Teeth 43 and 44, whereas the overlying mucosa appeared of normal color and showed no signs of inflammation (Figure 1). All adjacent teeth were vital, without abnormal mobility or endodontic symptoms. Another panoramic radiography was performed and showed a well‐circumscribed unilocular radiolucent lesion with a thin sclerotic margin, measuring approximately 16 × 12 mm, located between Teeth 43 and 44 (Figure 2). CBCT confirmed the presence of a radiolucent lesion associated with widening of the interradicular space and displacement of the roots of the involved teeth. Discontinuity and erosion of the buccal cortical plate were evident, whereas the lingual cortical plate appeared thinned but preserved (Figure 3a,b). Given the close relationship of the lesion to the apical regions of Teeth 43 and 44, its interradicular extension, and the anticipated need for complete surgical access and debridement in the apical area, a multidisciplinary treatment plan was established. Although the adjacent teeth were vital and asymptomatic at presentation, preoperative endodontic treatment of Teeth 43 and 44 was performed by the patient′s general dentist, together with professional scaling, in preparation for surgery (Figure 4). Following completion of these procedures, surgical enucleation of the lesion was scheduled. Preoperative management included antibiotic prophylaxis with amoxicillin/clavulanic acid (875/125 mg; two tablets 1 h before surgery), ibuprofen 600 mg administered 1 h before the procedure, and mouth rinses with 0.2% chlorhexidine gluconate three times daily starting the day before surgery.

**Figure 1:**
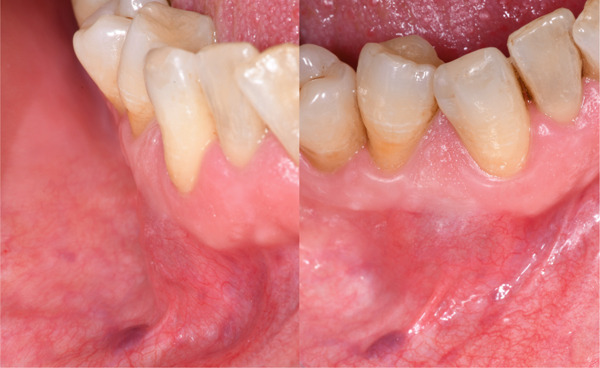
Intraoral clinical photograph of the lesion.

**Figure 2:**
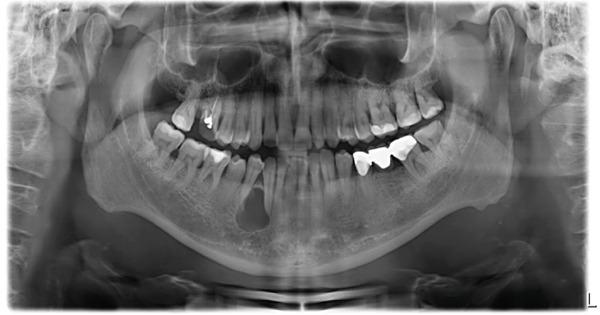
Baseline panoramic radiograph (T0).

**Figure 3:**
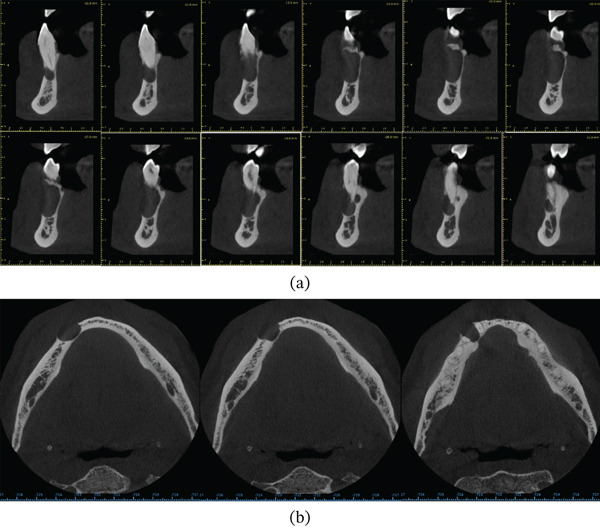
(a) Baseline CBCT cross‐sectional images (T0). (b) Baseline CBCT axial images (T0).

**Figure 4:**
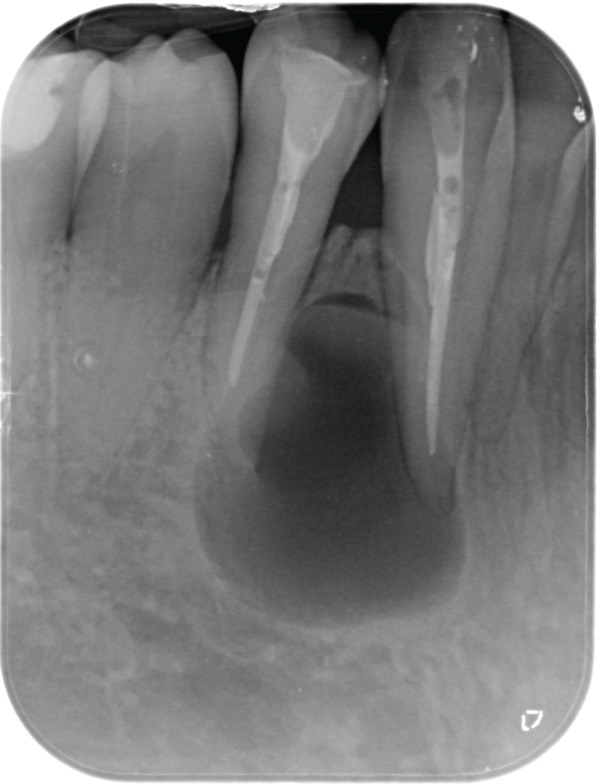
Periapical radiograph of the involved teeth (4.3–4.4) after completion of root canal treatment.

### 2.1. Surgical Procedure

The procedure was performed under local anesthesia using an inferior alveolar nerve block with mepivacaine without epinephrine, supplemented by local infiltrations with articaine 1:100,000. A marginal incision was extended from the distal aspect of Tooth 45 to the mesial aspect of Tooth 42, with two vertical releasing incisions to ensure adequate surgical access and to minimize the risk of flap tearing. A full‐thickness mucoperiosteal flap was elevated, exposing the mandibular canine–premolar region. The lesion was identified and carefully dissected from the surrounding bone using dedicated surgical instruments, allowing complete enucleation. After the removal of the cystic lesion, thorough mechanical debridement and curettage of the bone cavity were performed. Apicectomy of the adjacent teeth was subsequently carried out, followed by retrograde root‐end filling with mineral trioxide aggregate (MTA; Auroseal M.T.A.). Root‐end resection and retrograde filling were performed as part of the planned surgical management, given the lesion′s close proximity to the adjacent root apices and the need to ensure complete curettage/debridement of the apical and interradicular component. The surgical site was copiously irrigated with sterile saline solution. Given the CBCT and intraoperative evidence of buccal cortical plate discontinuity and erosion, interradicular extension, and reduced bony containment of the residual defect, guided bone regeneration (GBR) was performed as an adjunctive reconstructive measure. A xenogeneic bone graft (Bio‐Oss) was placed within the defect and covered with a resorbable collagen membrane (Evolution, Osteobiol), which was stabilized with titanium fixation tacks to support space maintenance, reduce soft tissue collapse, and facilitate bone fill during healing. Primary wound closure was achieved using 6‐0 polyamide monofilament sutures, applying vertical mattress stitches at the interdental papillae and simple interrupted sutures at the releasing incisions. The surgical specimen was fixed in formalin and submitted for histopathological examination.

### 2.2. Postoperative Management

Postoperative instructions emphasized meticulous oral hygiene to support uneventful healing. The patient was informed about expected postoperative symptoms, including swelling, pain, hematoma, and localized discomfort. Pharmacological management consisted of amoxicillin–clavulanate (875/125 mg) every 8 h for 5 days, along with analgesic/anti‐inflammatory therapy with ibuprofen (600 mg) as needed (maximum three tablets per day, taken after meals). In addition, the patient was instructed to perform mouth rinses with 0.2% chlorhexidine gluconate three times daily for 1 min using the undiluted solution, avoiding further rinsing, eating, or drinking for at least 1 h thereafter.

### 2.3. Histopathological Findings

Gross examination revealed a whitish soft tissue fragment consistent with a cyst wall, measuring approximately 1.5 cm in its greatest dimension. Histological analysis detected a multilocular odontogenic cystic lesion lined by odontogenic epithelium. The epithelial lining exhibited focal hyperplasia, papillary projections, and intraepithelial gland‐like (ductal) structures, with occasional clear cells. A mild chronic inflammatory infiltrate was present within the fibrous connective tissue wall. Although the lesion appeared predominantly unilocular on radiographic imaging, histopathological examination showed a multilocular botryoid architecture, confirming the diagnosis of BOC. This discrepancy is not unexpected, as BOC may appear radiographically as either a unilocular or multilocular radiolucency and may overlap with other odontogenic lesions on imaging.

### 2.4. Postoperative Course and Follow‐Up

The postoperative course was uneventful, and no complications were reported. At the 6‐month follow‐up, the patient was re‐evaluated at the Oral Surgery Unit, where a panoramic radiograph showed progressive bone healing (Figure 5). At 12 months, radiographic assessment performed by the patient′s general dentist revealed near‐complete bone regeneration within the surgical site (Figure 6). At the 24‐month follow‐up, complete bone fill was observed, with no clinical or radiographic evidence of recurrence (Figure 7a,b). At the time of manuscript preparation, 24 months represented the most recent available follow‐up interval; continued periodic clinical and radiographic surveillance is ongoing.

**Figure 5:**
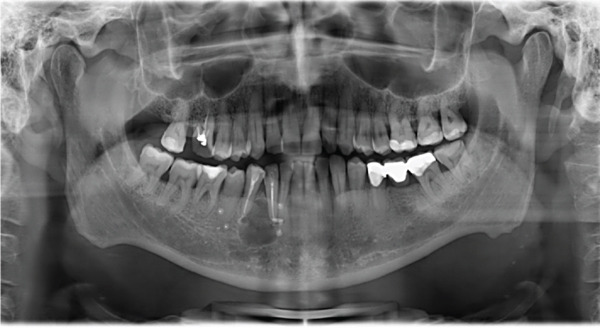
Six‐month follow‐up panoramic radiograph showing the extent of bone healing.

**Figure 6:**
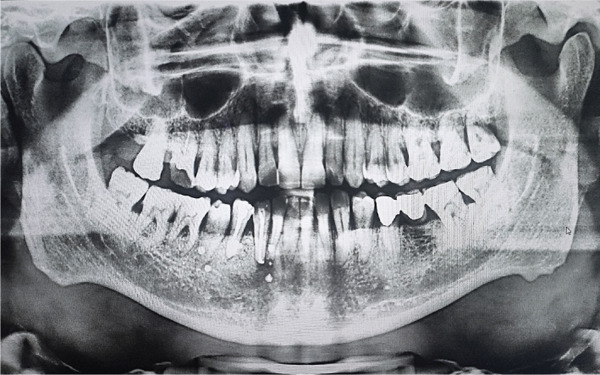
Twelve‐month follow‐up panoramic radiograph showing the near‐complete bone healing.

**Figure 7:**
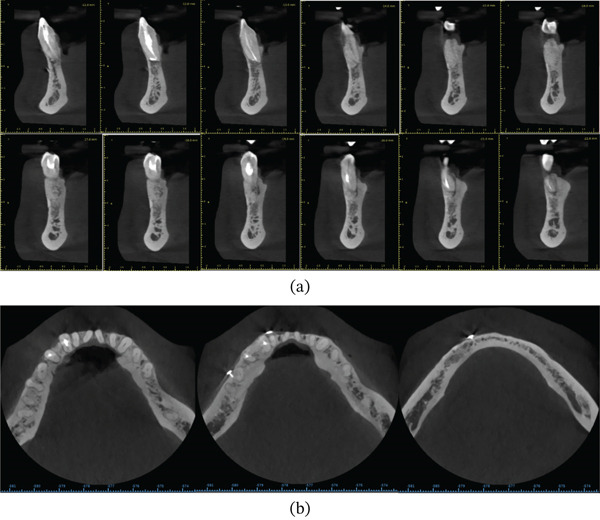
CBCT findings at 24‐month follow‐up showing satisfactory bone healing: (a) cross‐sectional images and (b) axial images.

## 3. Discussion

### 3.1. Clinicopathologic Features and Comparison With the Literature

BOC represents an uncommon multilocular variant of the LPC, characterized by a more expansile growth pattern and greater morphological complexity [1, 14]. Its clinical course is typically asymptomatic or only mildly symptomatic; therefore, diagnosis is often incidental, with subtle manifestations such as tooth displacement or a limited buccal swelling.

In the present case, the lesion was identified during routine radiographic assessment, in line with previous reports indicating that the majority of BOCs are diagnosed in the absence of overt clinical symptoms [15]. This highlights the importance of radiographic screening in the early detection of developmental odontogenic cysts. Radiographically, BOC typically presents a well‐defined unilocular or multilocular radiolucency, often without root resorption [16, 17]. Although these features are not pathognomonic, regular margins and internal septations may assist in the preliminary differential diagnosis. Nevertheless, radiographic findings may overlap with other odontogenic lesions, including odontogenic keratocysts and unicystic ameloblastomas, making histopathological examination essential for definitive diagnosis [11]. In the present case, the lesion appeared predominantly unilocular on CBCT, whereas histopathological examination showed a multilocular botryoid architecture; this discrepancy may occur when internal septa are thin or not appreciable on imaging. From an epidemiological perspective, BOC is most frequently reported in adults in the fifth decade of life and older, although cases in younger patients have also been described. In the large case series reported by Vidaković et al. [3], the mean age at diagnosis was 50.2 years (range 20–80). The same study identified a marked predilection for the mandible, accounting for the 83.3% of cases, which is consistent with the anatomical localization observed in the present report.

Regarding management, complete surgical enucleation remains the treatment of choice for small‐ to medium‐sized lesions. In more extensive cases, marsupialization or decompression has been proposed as a staged approach to reduce cyst volume prior to definitive enucleation. The choice of surgical technique should balance complete lesion removal with preservation of adjacent teeth and minimization of surgical morbidity [18].

Reported recurrence rates for BOC are variable but consistently nonnegligible, with some series describing rates approaching approximately 30%. Recurrence is generally attributed to the multilocular architecture of the lesion and the potential for incomplete removal of internal septa or residual epithelial remnants, rather than to intrinsically aggressive biological behavior [12]. For this reason, meticulous surgical technique and long‐term radiographic follow‐up are recommended.

### 3.2. Bone Defect Management After Enucleation: Spontaneous Healing Versus GBR

The indication for bone grafting after cyst enucleation remains debated and should be individualized. Several studies have shown that spontaneous bone regeneration may occur uneventfully after enucleation of jaw cysts, including relatively large defects, particularly when sufficient bony walls and periosteal support are preserved and primary closure is achieved [19–21]. In these situations, grafting may be unnecessary and may increase cost and procedural complexity. Conversely, immediate regenerative management may be considered in selected defects with reduced bony containment (such as cortical plate loss or discontinuity), periodontal involvement, or when space maintenance and soft tissue support are clinically relevant. Case reports on LPCs and related developmental odontogenic cysts have described favorable outcomes after enucleation combined with GBR when defect morphology was considered unfavorable for spontaneous contour restoration alone [22–24]. In the present case, GBR was not intended as a routine step for BOC management but as a case‐specific adjunctive measure. The lesion measured approximately 16 × 12 mm on panoramic radiography and was associated on CBCT with interradicular extension, buccal cortical plate discontinuity and erosion, and reduced defect containment, whereas the lingual cortical plate was thinned but preserved. Intraoperative findings confirmed the buccal cortical defect. For these reasons, GBR was used as an adjunctive measure rather than as a routine step in BOC management. The use of a xenogeneic graft and resorbable membrane was intended to support space maintenance, reduce soft tissue collapse, and facilitate bone fill. Radiographic follow‐up at 6 and 12 months (panoramic radiographs) and at 24 months (CBCT) showed progressive bone regeneration with no evidence of recurrence. Although this outcome is favorable, recurrence of BOC may occur beyond 24 months. The 24‐month time point reflects the current duration of observation at the time of manuscript preparation rather than a planned cessation of surveillance. Continued long‐term clinical–radiographic follow‐up is ongoing and remains advisable in view of the reported risk of late recurrence.

Overall, BOC requires an accurate diagnostic workflow and a carefully planned surgical approach integrating three‐dimensional imaging, histopathological confirmation, and long‐term clinical–radiographic surveillance.

### 3.3. Limitations

The present report has some limitations. First, as a single case report, the findings cannot be generalized and should be interpreted with caution. Second, although the histopathological diagnosis was available, the original histological slide could not be retrieved from the Pathology Department despite repeated requests, limiting the possibility of providing direct microscopic documentation. Third, the use of GBR should not be interpreted as a standard treatment for BOCs, but rather as a case‐specific adjunctive reconstructive measure adopted because of the buccal cortical plate loss, reduced bony containment, and need for soft tissue support. Finally, although no clinical or radiographic evidence of recurrence was observed at 24 months, longer follow‐up remains necessary because late recurrence of BOCs has been reported.

## 4. Conclusions

BOC is a rare developmental odontogenic cyst that warrants a thorough diagnostic workup with histopathological confirmation, given its overlap with other radiolucent jaw lesions and its risk of recurrence. Complete surgical enucleation remains the preferred treatment; however, due to its multilocular pattern, a structured radiographic follow‐up is advisable, with postoperative assessments at 6 and 12 months and then annually for at least 5 years, using intraoral periapical radiographs and CBCT when healing is uncertain or recurrence is suspected.

## Author Contributions

G.P. and P.F. performed the diagnosis and surgical intervention. M.S. and P.S. drafted the original manuscript. P.S. performed the clinical photography. S.T. and C.B. contributed to manuscript revision and provided critical feedback.

## Funding

Open access publishing facilitated by Universita degli Studi di Bologna, as part of the Wiley ‐ CRUI‐CARE agreement.

## Ethics Statement

According to institutional policy, formal ethics committee approval is not required for single case reports.

## Consent

The patient has been adequately informed about the treatment, understands the risks and benefits, and consents to proceed. The patient also consents to the use of photographs and radiographic exams for scientific and educational purposes, authorizing their anonymous publication. The informed consent has been signed.

## Conflicts of Interest

The authors declare no conflicts of interest.

## Data Availability

The data used to support the findings of this study are available from the corresponding author upon request.
